# Psychological Impact of Political Conflict: Prevalence and Severity of PTSD Among Children and Adolescents in the North and Middle West Bank Following the October 7, 2023, Events

**DOI:** 10.1155/nrp/7304673

**Published:** 2025-08-06

**Authors:** Ibrahim Aqtam, Rawan Naghnaghiyeh, Taqwa Zubaide, Yusor Othman, Yasser Malasah

**Affiliations:** Department of Nursing, Ibn Sina College for Health Professions, Nablus University for Vocational and Technical Education, Nablus, State of Palestine

**Keywords:** adolescents, children, CPSS scale, political violence, PTSD, trauma, West Bank, W-TEC

## Abstract

**Background:** The political conflict in the West Bank was escalated on October 7, 2023, further increasing exposure to traumatic events among children and adolescents, with potential implications for their mental health. This study estimates the prevalence and severity of posttraumatic stress disorder (PTSD), its sociodemographic correlates, and functional impacts on affected youth.

**Methods:** This cross-sectional study was conducted from February 5 to March 5, 2024, among 1148 Palestinian children and adolescents aged 8–15 years from the North and Middle West Bank, selected by stratified random sampling. The Child PTSD Symptom Scale was used to assess symptoms of PTSD, while trauma exposure was measured using the War-Trauma Exposure Checklist. SPSS was used for data analysis.

**Results:** Symptoms of PTSD were moderate to very severe in 70.0% of participants. The most prevalent traumatic experiences were witnessing bombings on television (93.4%) and witnessing dismembered bodies on television (92.8%). Intrusion symptoms predominated, with frequent reports of nightmares and intrusive thoughts. Age showed a significant positive correlation with PTSD severity (*r* = 0.12, *p*=0.034), indicating more severe symptoms in older children. PTSD significantly impaired academic performance (55.0%), emotional relationships (41.5%), and recreational activities (32.7%). Children experiencing > 5 traumatic events had substantially higher rates of severe PTSD symptoms (73.6% of this group; *p*=0.003).

**Conclusions:** These findings indicate a severe mental health crisis among conflict-exposed children in the West Bank. Immediate interventions are needed, including trauma-focused cognitive behavioral therapy delivered with substantial modifications for ongoing conflict settings and school-based mental health programs. Respite for children and parents may be an important first step to engaging children and families in therapy. Systemic barriers to mental health care need to be tackled if better outcomes are achieved in this vulnerable population.

## 1. Introduction

The protracted Israeli-Palestinian conflict has left an indelible mark on the mental health of Palestinian children and adolescents. Being one of the most vulnerable populations, these young individuals face heightened exposure to war-related trauma. On October 7, 2023, Hamas launched a large-scale attack on Israel, triggering sustained military operations across the West Bank. This escalation resulted in unprecedented violence in the North and Middle regions, including intensified airstrikes, ground incursions, mass arrests, and displacement of civilians—creating new layers of trauma for children [1].

Posttraumatic stress disorder (PTSD) is a debilitating mental health condition characterized by intrusive memories, avoidance behaviors, cognitive distortions, and hyperarousal that severely impair functioning [2]. Children in conflict zones show alarmingly high PTSD rates (25%–70%) depending on exposure intensity [3, 4], with symptoms manifesting as academic decline, social withdrawal, sleep disturbances, and behavioral problems [5, 6]. In Palestine specifically, continuous conflict and socioeconomic hardships compound mental health challenges, while limited-service access creates critical treatment gaps [7].

Sociodemographic factors influence PTSD vulnerability, though findings show inconsistencies. Some studies associate older age, female gender, and urban residence with higher risk [8–10], while others report nonsignificant effects of gender and age [11–13]. These discrepancies highlight the presence of context-dependent mediators that require localized investigation.

Traumatic events like witnessing violence, bereavement, or displacement are established PTSD triggers [14]. Recent Syrian research identified family member deaths and home destruction as particularly devastating [15], while West Bank studies link military operations to symptom exacerbation [16]. Evidence-based interventions like trauma-focused cognitive behavioral therapy (TF-CBT) show efficacy in post-conflict settings [17, 18], though substantial adaptations are needed during ongoing violence [19] and therapeutic respite may facilitate engagement [20].

This study addresses critical gaps by assessing PTSD prevalence and severity following the October 2023 escalation, analyzing associations with age, gender, and residence, examining functional impairments across academic, social, and familial domains, and investigating trauma type–dose relationships. The findings will directly inform school-based mental health programs and adapted clinical interventions tailored to the ongoing crisis context [21].

## 2. Methods

### 2.1. Study Design

The present study used a descriptive cross-sectional design to estimate the prevalence and severity of symptoms of PTSD among Palestinian children and adolescents aged 8–15 years in the North and Middle West Bank. It also examined the association between PTSD symptoms with sociodemographic variables and the impact of PTSD on functional domains like academic performance and social relationships. The War-Trauma Evaluation Checklist (W-TEC) was adapted to reflect the unique traumatic experiences related to the October 7, 2023, escalation by adding context-specific items, such as witnessing bombings and displacement during the military events, while retaining the validity and reliability of the tool. Modifications were confirmed through a pilot study for relevance and cultural appropriateness.

### 2.2. Population and Sample Size

The study population consisted of children and adolescents attending schools in the governorates of Jenin, Tulkarm, and Ramallah. Participants were distributed across age levels as follows: 9.4%–14.6% from each age level between 8 and 15 years. The distribution of participants across these governorates was informed by population size and the intensity of conflict exposure in each area, with Jenin experiencing higher levels of violence and displacement compared to Tulkarm and Ramallah [22]. The sample size calculation was determined to be representative, using the Raosoft sample size calculator. It estimated a sample size of 1187 participants for a 95% confidence level and 5% margin of error. Proportional stratified sampling was adopted to distribute participants as follows: Jenin, 37% (*n* = 425); Tulkarm, 23.9% (*n* = 274); and Ramallah, 39.1% (*n* = 449). These percentages thus reflected the respective populations in each governorate and their various exposures to conflict. Allowing for nonresponses, a total of 1200 questionnaires were distributed. Of these, 1148 were completed, yielding an extremely high response rate of 95.7%. This strong response rate increases the robustness and representativeness of the results.

### 2.3. Sampling Method

Schools were randomly selected from each governorate, including urban, rural, and refugee camp settings. In each school, participants were selected by a simple random sampling method. Students with previous diagnoses of mental health problems or outside of the age range of 8–15 years were excluded [23].

### 2.4. Data Collection

The collection of data occurred between February 5 and March 5, 2024. For younger participants aged 8–12 years, the interview had to be structured, keeping in mind limitations concerning literacy and comprehension. Standardized questions were used, adapted from the W-TEC and CPSS tools to ensure consistency and reliability. Interviewers were trained in minimizing bias and using a child-friendly approach. The questionnaires were self-completed by the older participants (13–15 years) under supervised conditions to ensure privacy and minimize inaccuracies. Potential biases due to self-reporting were minimized by explaining anonymity and confidentiality of responses to reduce social desirability bias. In addition, participants were given sufficient time to complete the questionnaires and were encouraged to ask for clarification where necessary.

### 2.5. Instruments

Two validated tools were used for data collection.

1. W-TEC: This 22-item checklist was adapted for events following October 7, 2023, and captures participants' levels of exposure to war-related traumatic experiences through yes/no responses. The W-TEC demonstrates strong validity (*κ* = 0.89) in conflict settings [24]. The checklist assesses various categories of trauma including personal injuries, witnessing violence, and witnessing property destruction. A panel of three clinical psychologists and two trauma researchers independently reviewed all 22 W-TEC items for clinical relevance and prevalence (> 30% threshold). Consensus was achieved through iterative discussion until unanimous agreement on the 8 most significant items. Only 8/22 items are reported in the results section due to clinical relevance and > 30% prevalence, representing the most prevalent and clinically significant traumatic events experienced by the study population [24–26].
2. CPSS: a 27-item scale measuring PTSD symptoms according to DSM-5 criteria. The first 20 items evaluate symptom severity across four categories: reexperiencing, avoidance, negative alterations in cognition and mood, and hyperarousal. The remaining 7 items assess functional impairment in various domains including school performance, relationships with friends, family relationships, and general daily functioning. Each impairment item is rated on a 4-point scale (0 = not at all, 1 = little bit, 2 = pretty much, and 3 = very much). The total symptom scores range from 0 to 80, with severity categorized as: minimal (0–10), mild (11–20), moderate (21–40), severe (41–60), and very severe (61–80) [27]. The CPSS demonstrated high internal consistency in this study with Cronbach's alpha = 0.851.

### 2.6. Ethical Considerations

This study received approval from the Institutional Review Board (IRB) of Nablus University for Vocational and Technical Education (Ref: Nur.Feb.2024/2). The study followed the principles of the Declaration of Helsinki on ethical research involving human participants. Informed consent was obtained from the parents; assent was obtained from the participants. Participants were assured of confidentiality, and all were informed of their right to withdraw from the study at any time without penalty.

### 2.7. Data Analysis

Data analysis was conducted using SPSS Version 23 [28]. Descriptive statistics were used to summarize demographic characteristics, trauma exposure, and PTSD severity. Pearson's correlation, ANOVA, and chi-square tests were conducted to assess associations between PTSD symptoms and trauma exposure with sociodemographic variables. ANOVA compared PTSD severity across age groups (8–10, 11–13, and 14–15 years) to examine developmental differences in trauma responses.

## 3. Results

### 3.1. Participant Characteristics

In all, 1148 Palestinian children and adolescents aged 8–15 years participated in the study. The mean age of participants was 11.5 years (SD = 2.1), with a nearly equal gender distribution of 50.8% male and 49.2% female. Geographically, participants were distributed across Jenin (37.0%), Tulkarm (23.9%), and Ramallah (39.1%), with representation from cities (35%), villages (38.5%), and refugee camps (26.5%).

### 3.2. Exposure to Traumatic Events

All participants reported at least one war-related traumatic event. The most prevalent war-related traumatic events were watching homes and people being bombed on television (93.4%) and watching dismembered bodies and injured people on television (92.8%). Other frequently reported events included inhaling tear gas (65.3%) and witnessing a relative being shot (32.1%). The least frequently reported events included being used as a human shield by the Israeli army (9.5%) and being arrested (13.1%) (Table 1). Only 8/22 items are reported due to clinical relevance and > 30% prevalence threshold, representing the most significant traumatic exposures in this population.

### 3.3. Prevalence and Severity of PTSD Symptoms

Most participants (70.0%) exhibited moderate to very severe PTSD symptoms, with 38.8% showing moderate symptoms, 22.0% severe symptoms, and 9.3% very severe symptoms. Only 7.1% showed minimal symptoms, while 22.8% exhibited mild symptoms (Table 2).

### 3.4. Types of PTSD Symptoms

Symptoms of PTSD were divided into four domains: intrusion, avoidance, negative alterations in cognition and mood, and hyperarousal. Intrusion symptoms predominated (*M* = 15.2, SD = 5.1), followed by hyperarousal symptoms (*M* = 12.6, SD = 4.8). Avoidance symptoms had a mean score of 9.8 (SD = 4.2), while negative alterations in cognition and mood had a mean score of 11.4 (SD = 5.3). The prominence of intrusion symptoms indicates that reexperiencing trauma through nightmares and intrusive thoughts was the most distressing manifestation for participants.

### 3.5. PTSD Impact on Daily Functioning

PTSD was defined as a dichotomous variable by grouping minimal, mild, and moderate symptoms as “low PTSD symptoms” and severe and very severe symptoms as “high PTSD symptoms.” The analysis revealed that PTSD symptoms significantly impaired participants' daily activities across multiple domains. Academic performance was the most affected area, with impairment reported in 55.0% of participants, followed by emotional relationships (41.5%), recreational activities (32.7%), and family interactions (24.6%) (Table 3).

### 3.6. Sociodemographic Correlates of PTSD Symptoms

Sociodemographic variables of age, gender, and residence were analyzed for their association with PTSD symptom severity using Pearson correlation, chi-square tests, and ANOVA. There was a weak but significant correlation between age and PTSD severity (*r* = 0.12, *p*=0.034), with older children reporting higher symptom levels. Gender differences were not significant (*p*=0.083). Children from urban areas reported significantly higher PTSD symptoms than those in rural areas or camps (*p*=0.021) (Table 4).

### 3.7. Association of Traumatic Events With Symptoms of PTSD

A strong association existed between traumatic event frequency and PTSD severity (*p*=0.003). Children experiencing > 5 traumatic events had significantly higher rates of severe/very severe symptoms (73.6%). Witnessing televised bombings (*r* = 0.23, *p*=0.002), dismembered bodies (*r* = 0.21, *p*=0.004), and inhaling tear gas (*r* = 0.18, *p*=0.005) showed the strongest symptom correlations. Being arrested (*r* = 0.15, *p*=0.007) and human shield exposure (*r* = 0.13, *p*=0.027) were also significant predictors (Table 5).

## 4. Discussion

For the first time, on a large representative sample, the current study disclosed and analyzed the serious psychological impact of political conflict on children and adolescents in the North and Middle West Bank following the events of October 7, 2023. Extremely high rates of PTSD symptomatology were documented, together with functional impairments and significant dose–response relationships between traumatic experiences and PTSD severity. Our findings provide important insights into how youth endure the effects of war situations, calling for clearly targeted policies and interventions.

The study found that 70% of participants had moderate to very severe PTSD symptoms, in agreement with findings from studies conducted in other conflict-prone areas. For example, a 2021 study in Afghanistan reported that 65% of children exposed to war-related violence qualified for PTSD [29]. This similarity reinforces the devastating psychological effects of prolonged exposure to conflict, especially among youth in politically unstable regions. However, a study in South Sudan found a lower PTSD prevalence of 40% among adolescents, attributing the difference to variations in cultural resilience and social support systems [30]. These discrepancies suggest that regional sociocultural factors may mediate the psychological impact of conflict, warranting further exploration in the West Bank context.

Intrusion symptoms were the most reported in this study, especially recurring thoughts and nightmares. This finding agrees with research conducted in Ethiopia on the prevalence of intrusion symptoms among children exposed to violence during armed conflict [31]. These symptoms suggest that trauma is pervasive and intrusive, and that therapeutic interventions need to be implemented to address these specific manifestations of PTSD.

Age was a significant covariate, with older children reporting higher symptom severity. This corresponds with results from a study in Yemen where adolescents had higher PTSD symptoms than younger children, possibly due to better understanding of the implications of conflict and longer exposure to violence [32]. In contrast, no significant relationship between age and PTSD symptom severity was identified in research among school children in Nigeria, suggesting that developmental stage is not universally a mediator of trauma responses [33]. These divergent findings underscore the importance of localized research in understanding age-specific dynamics of PTSD in conflict settings.

Although this study did not find statistically significant gender differences, this contrasts with findings from a study in Myanmar where females had higher PTSD rates, partly due to gender-specific vulnerabilities, including gender-based violence [15]. This points to the complexity of gendered experiences of trauma and the need for nuanced analysis of gender-specific factors in future studies.

In the current study, urban children reported higher levels of PTSD symptoms than their rural and refugee camp counterparts. Similarly, in Colombia, urban youth exposed to gang violence and urban conflict had higher rates of PTSD than rural youth [34]. This may be explained by the intensity of violence in urban settings with high population density, which increases the psychological impact of trauma. Higher urban PTSD suggests targeted outreach in densely populated areas, where mental health services should be concentrated and community-based interventions prioritized to reach the most affected populations efficiently.

PTSD symptoms significantly disrupt academic performance (55%) and social relationships (41.5%). These findings agree with a study conducted in Sierra Leone, in which PTSD was associated with impaired cognitive functioning and lower school attendance in war-exposed children [35]. Similarly, social withdrawal and interpersonal problems were common among children with PTSD in Uganda, consistent with the relational disturbances found in this study [36]. Such impairments reflect the pervasive impact of trauma on children's development and underscore the need for school-based mental health interventions.

The number of trauma types and PTSD severity showed statistical significance. Individuals exposed to more than five traumatic events were more likely to show severe symptoms, consistent with results from a study conducted in Lebanon where cumulative trauma exposure was strongly related to PTSD severity [37]. However, one study conducted in Kenya found that the nature rather than the number of traumatic events accounted more for PTSD symptom severity, which may indicate that specific types of traumas have disproportionate impact [38]. This inconsistency underlines the need for detailed assessments of trauma characteristics in future research.

### 4.1. Strengths and Limitations

The strengths of this study include its large, representative sample and the use of validated tools adapted for the specific context. However, reliance on self-reported data may introduce potential response bias, and the cross-sectional design limits causal inferences. Future studies should adopt longitudinal approaches to capture the long-term effects of trauma and evaluate the efficacy of interventions.

### 4.2. Implications for Practice and Policy

These findings indicate the urgent need for evidence-based mental health interventions for children and adolescents affected by conflict. Although TF-CBT has been shown to benefit children in the aftermath of violent conflicts, when delivered in the context of ongoing conflicts, substantial modifications may be necessary and respite for children and parents may be an important first step to engaging children and families in therapy [19, 20]. In addition, integrating mental health services into schools and community centers provides accessible support for affected youth. Equitable access to care would involve addressing barriers to accessing mental health services, especially in rural areas.

## 5. Conclusion

This study demonstrates the extent of the psychological impact of political conflict on children and adolescents in the West Bank. Understanding trauma exposure, sociodemographic factors, and functional impairments may help mitigate the long-term impact of violence on this vulnerable population and enhance resilience through targeted interventions and policies.

## Figures and Tables

**Table 1:** Prevalence of selected traumatic events (*N* = 1148).

Traumatic event	Yes (*n*)	Yes (%)	No (*n*)	No (%)
Witnessing a relative being shot	369	32.1	779	67.9
Witnessing a friend being shot	281	24.5	867	75.5
Inhaling tear gas	750	65.3	398	34.7
Witnessing neighbors' homes being demolished	245	21.3	903	78.7
Watching homes and people bombed on TV	1072	93.4	76	6.6
Watching dismembered bodies on TV	1065	92.8	83	7.2
Being used as a human shield	111	9.5	1037	90.5
Being arrested	150	13.1	998	86.9

**Table 2:** Severity of PTSD symptoms (*N* = 1148).

Severity level	Frequency (*n*)	Percentage (%)
Minimal (0–10)	82	7.1
Mild (11–20)	262	22.8
Moderate (21–40)	446	38.8
Severe (41–60)	252	22.0
Very severe (61–80)	106	9.3

**Table 3:** Impact of PTSD on daily functioning (*N* = 1148).

Domain affected	Frequency (*n*)	Percentage (%)
Academic performance	631	55.0
Emotional relationships	476	41.5
Recreational activities	376	32.7
Family interactions	282	24.6

**Table 4:** PTSD symptoms by sociodemographic variables.

Variable	Mean PTSD score (SD)	*p* value
*Age group*		
8–10 years	26.8 (12.5)	0.034
11–13 years	32.4 (14.2)	
14–15 years	35.6 (15.8)	

*Gender*		0.083
Male	30.2 (13.9)	
Female	31.5 (14.4)	

*Place of residence*		0.021
City	33.8 (14.7)	
Village	29.7 (13.8)	
Refugee camp	28.9 (12.4)	

**Table 5:** PTSD severity by number of traumatic events (*N* = 1148).

Number of traumatic events	Minimal/mild PTSD (*n*, %)	Moderate PTSD (*n*, %)	Severe/very severe PTSD (*n*, %)	*p* value
1–3	92 (35.9%)	68 (15.2%)	28 (8.4%)	*p*=0.003
4–6	108 (42.2%)	156 (34.9%)	84 (25.0%)	
7+	44 (21.9%)	222 (49.8%)	246 (73.6%)	

## Data Availability

The datasets generated and analyzed during the current study are available from the corresponding author upon reasonable request.

## References

[1] American Psychiatric Association (2013). *Diagnostic and Statistical Manual of Mental Disorders*.

[2] World Health Organization (2021). *Mental Health and Psychosocial Well-Being of Children Exposed to Armed Conflict*.

[3] UNICEF (2020). *Children and Armed Conflict. Annual Report of the Secretary-General*.

[4] Slone M., Shoshani A. (2016). The Impact of Trauma Exposure on the Mental Health of Children in Conflict Zones: A Risk and Resilience Perspective. *Journal of Child Psychology and Psychiatry*.

[5] Giacaman R., Khatib R., Shabaneh L. (2009). Health Status and Health Services in the Occupied Palestinian Territory. *The Lancet*.

[6] Alisic E., Jongmans M. J., van Wesel F., Kleber R. J. (2011). Children’s Perspectives on Dealing With Trauma: A Qualitative Meta-Analysis. *Clinical Child and Family Psychology Review*.

[7] Thabet A. M., Vostanis P. (2011). Impact of Trauma on Palestinian Children’s Mental Health in the Gaza Strip and the West Bank. *The Lancet*.

[8] Pat-Horenczyk R., Qasrawi R., Leshem B., Haj-Yahia M. M. (2009). Posttraumatic Distress in Palestinian Adolescents. *Child Abuse and Neglect*.

[9] Khamis V. (2005). Post-Traumatic Stress and Psychiatric Disorders in Palestinian Adolescents Exposed to Violence in the West Bank. *Journal of Traumatic Stress*.

[10] Barber B. K. (2001). Political Violence, Social Integration, and Youth Functioning: Palestinian Youth From Different Political and Social Contexts. *International Journal of Behavioral Development*.

[11] Elbedour S., Onwuegbuzie A. J., Ghannam J., Whitcome J. A., Abu Hein F. (2007). The Impact of Exposure to Violence on PTSD, Depression, and Anxiety in Children From Gaza. *Journal of Loss and Trauma*.

[12] Betancourt T. S., Newnham E. A., Layne C. M. (2012). Trauma History and Psychosocial Adjustment in War-Affected Youth: Examining Pathways to Resilience. *Journal of Adolescent Health*.

[13] Miller K. E., Rasmussen A. (2010). War Exposure, Daily Stressors, and Mental Health in Conflict and Post-Conflict Settings: Bridging the Divide Between Trauma-Focused and Psychosocial Frameworks. *Social Science and Medicine*.

[14] Thabet A. M., Karim K., Vostanis P. (2006). Trauma Exposure in Pre-School Children in a War Zone. *The British Journal of Psychiatry*.

[15] Catani C., Jacob N., Schauer E., Kohila M., Neuner F. (2008). Family Violence, War, and Natural Disasters: A Study of the Effect of Extreme Stress on Children’s Mental Health in Sri Lanka. *BMC Psychiatry*.

[16] Giacaman R., Shannon H. S., Saab H., Arya N., Boyce W. (2007). Individual and Collective Exposure to Political Violence: Palestinian Adolescents Coping With Conflict. *Social Science and Medicine*.

[17] Cohen J. A., Mannarino A. P., Iyengar S. (2011). Community Treatment of Posttraumatic Stress Disorder for Children Exposed to Intimate Partner Violence: A Randomized Controlled Trial. *Archives of Pediatrics and Adolescent Medicine*.

[18] Betancourt T. S., Williams T., Kellner S. E., Gebre-Medhin J., Hann K., Kayiteshonga Y. (2014). A Behavioral Intervention for War-Affected Youth in Sierra Leone. *Journal of the American Academy of Child and Adolescent Psychiatry*.

[19] Pfeiffer E., Beer R., Birgersson A. (2023). Implementation of an Evidence-Based Trauma-Focused Treatment for Traumatised Children and Their Families During the War in Ukraine: A Project Description. *European Journal of Psychotraumatology*.

[20] Ford J. D. (2025). Responding to the Need for Psychotherapeutic Respite by War-Exposed Ukrainian Children. *JAMA Network Open*.

[21] American Psychological Association (2014). *The Road to Resilience*.

[22] Palestinian Central Bureau of Statistics (2023). Demographic Survey of the West Bank and Gaza Strip. *Ramallah, Palestine*.

[23] Etikan I., Musa S. A., Alkassim R. S. (2016). Comparison of Convenience Sampling and Purposive Sampling. *American Journal of Theoretical and Applied Statistics*.

[24] Smith P., Perrin S., Yule W., Hacam B., Stuvland R. (2016). The War-Trauma Screening Checklist: A Practical Tool for Identifying Children Affected by War. *Journal of Child Psychology and Psychiatry*.

[25] Israelashvili M., Zelniker T. (2020). Assessing the Impact of Traumatic Events on Youth: Current Knowledge and Future Directions. *Trauma, Violence, and Abuse*.

[26] Martin C. R., Preedy V. R., Patel V. B. (2016). *Comprehensive Guide to Post-Traumatic Stress Disorders*.

[27] Foa E. B., Johnson K. M., Feeny N. C., Treadwell K. R. (2001). The Child PTSD Symptom Scale (CPSS): A Diagnostic and Severity Measure for Youth With Posttraumatic Stress Disorder. *Psychological Assessment*.

[28] IBM Corp (2015). *IBM SPSS Statistics for Windows, Version 23.0*.

[29] Panter-Brick C., Eggerman M., Mojadidi A., McDade T. W. (2008). Social Stressors, Mental Health, and Physiological Stress in an Urban Elite of Afghanistan. *Human Biology*.

[30] Betancourt T. S., Meyers‐Ohki S. E., Charrow A. P., Hansen N. (2013). Annual Research Review: Mental Health and Resilience in HIV/AIDS-Affected Children—A Review of the Literature and Recommendations for Future Research. *Journal of Child Psychology and Psychiatry*.

[31] Dadi A. F., Dachew B. A., Kisi T., Yigzaw N., Azale T. (2016). The Prevalence of Post-Traumatic Stress Disorder and its Predictors Among Child Survivors of Armed Conflict in Ethiopia. *BMC Psychiatry*.

[32] Adeyinka O., Baiyewu O. (2019). Prevalence and Correlates of Posttraumatic Stress Disorder Among Adolescents in Northern Nigeria. *BMC Psychiatry*.

[33] Bryant R. A., Creamer M., O’Donnell M., Silove D., McFarlane A. C. (2008). A Multisite Study of the Capacity of Acute Stress Disorder Diagnosis to Predict Posttraumatic Stress Disorder. *The Journal of Clinical Psychiatry*.

[34] Betancourt T. S., McBain R. K., Newnham E. A., Brennan R. T. (2015). The Intergenerational Impact of War: Longitudinal Relationships Between Caregiver and Child Mental Health in Post-Conflict Sierra Leone. *Journal of Child Psychology and Psychiatry*.

[35] Kohrt B. A., Jordans M. J. (2015). Resilience and Mental Health in Children and Adolescents Living in Areas of Armed Conflict—A Call for Innovative Intervention and Implementation Research. *Child Abuse and Neglect*.

[36] Karam E. G., Mneimneh Z. N., Dimassi H. (2008). Lifetime Prevalence of Mental Disorders in Lebanon: First Onset, Treatment, and Exposure to War. *PLoS Medicine*.

[37] Pynoos R. S., Steinberg A. M., Piacentini J. C. (1999). A Developmental Psychopathology Model of Childhood Traumatic Stress and Intersection With Anxiety Disorders. *Biological Psychiatry*.

[38] Cohen J. A., Deblinger E., Mannarino A. P., Steer R. A. (2004). A Multisite, Randomized Controlled Trial for Children With Sexual Abuse-Related PTSD Symptoms. *Journal of the American Academy of Child and Adolescent Psychiatry*.

